# Impact of globalisation on family relationships and dynamics as expressed by married adults in Lagos State, Nigeria

**DOI:** 10.3389/fsoc.2026.1834401

**Published:** 2026-05-04

**Authors:** Lateef Omotosho Adegboyega, OmoshalewaLasbat Akinsemoyin, Shukurat Modupe Oloyede

**Affiliations:** 1 Department of Educational Guidance and Counselling, Faculty of Education, University of Ilorin, Ilorin, Nigeria; 2 Department of Social Sciences Education, Faculty of Education, University of Ilorin, Ilorin, Nigeria; 3 Department of Adult and Primary Education, Faculty of Education, University of Ilorin, Ilorin, Nigeria

**Keywords:** family dynamics, family relationships, globalisation, Lagos State, married adults

## Abstract

**Introduction:**

Globalisation has transformed the way families interact, communicate, and navigate their relationships across contemporary societies. In Nigeria, particularly in urban centres such as Lagos State, increasing exposure to global economic, technological, and cultural influences has significantly reshaped family dynamics. This study investigated the impact of globalisation on family relationships and dynamics among married adults in Lagos State, Nigeria, with emphasis on communication patterns, role expectations, conflict experiences, and family cohesion.

**Methods:**

A mixed-methods approach was employed, integrating both quantitative and qualitative data collection and analysis. A total of 300 married adults were selected through a multi-stage sampling technique for the survey, while 30 participants were purposively selected for in-depth interviews. Quantitative data were analysed using descriptive statistics (mean and standard deviation) and inferential statistics (Pearson correlation and regression analysis), while qualitative data were analysed using thematic analysis.

**Results:**

The findings revealed that globalisation has both positive and negative impacts on family relationships and dynamics. Quantitatively, respondents agreed that globalisation enhances communication, promotes cultural understanding, and improves shared decision-making (grand mean = 3.54), while also contributing to reduced face-to-face interaction, increased work-related pressures, and value conflicts (grand mean = 3.22). Inferential analysis indicated a significant relationship between globalisation and family relationship dynamics (*r* = 0.62, *p* < 0.05). Regression results further showed that globalisation significantly predicts family relationship outcomes (*β* = 0.58, *p* < 0.05), accounting for 34% of the variance. Qualitative findings corroborated these results, revealing that married adults adopt coping strategies such as improved communication, role renegotiation, reliance on social support systems, and preservation of cultural values.

**Discussion and recommendations:**

The study concluded that globalisation plays a dual role in shaping family relationships, acting both as a facilitator of relational connectivity and a source of familial strain. While it enhances communication and access to resources, it also introduces challenges related to role conflict, reduced family interaction, and value dissonance. It is recommended that married adults, policymakers, and community stakeholders adopt strategies that promote effective communication, flexible role negotiation, and strengthened social support systems, alongside community-based initiatives aimed at sustaining family cohesion and resilience in a globalised society.

## Introduction

Globalisation has become one of the most influential forces shaping modern societies, transforming economic systems, cultural practices, social institutions, and interpersonal relationships across the world. In recent decades, families have experienced significant transformations due to the rapid expansion of global communication technologies, migration patterns, economic restructuring, and exposure to diverse cultural norms and values. These transformations have altered traditional family roles, communication patterns, and expectations among married couples. In developing countries such as Nigeria, where family structures have historically been rooted in collectivist values and strong kinship ties, the effects of globalisation are particularly noticeable. The increasing integration of Nigerian society into global networks has influenced how married adults interact, make decisions, and maintain family cohesion in contemporary society (Adeleke and Adebayo, 2022; Okeke and Ibrahim, 2023; Adeboye, 2024).

Globalisation has significantly reshaped communication patterns within families, particularly among married adults. Advances in digital technologies such as smartphones, social media platforms, and instant messaging applications have enabled spouses to maintain communication despite geographical separation. These technologies have facilitated real-time communication and increased emotional connectivity among family members. However, while digital communication enhances accessibility and frequency of interaction, it may also reduce face-to-face communication and emotional depth within marital relationships. Studies have shown that excessive reliance on digital communication can lead to misunderstandings, reduced emotional intimacy, and weakened relational bonds between spouses (Eze and Nwosu, 2022; Ogunleye and Ajayi, 2023; Chukwuemeka and Ojo, 2024). Consequently, globalisation presents both opportunities and challenges for maintaining healthy family relationships.

In addition to communication patterns, globalisation has also influenced gender roles and role expectations within Nigerian families. Traditionally, Nigerian families operated under patriarchal systems where men served as breadwinners while women were primarily responsible for domestic duties and child-rearing. However, globalisation has increased access to education and employment opportunities for women, leading to a shift in traditional family roles. As more women participate in the workforce, families increasingly adopt dual-income structures, which require couples to renegotiate household responsibilities and decision-making roles. While this shift promotes equality and shared responsibilities, it may also generate conflicts where traditional expectations persist. Research indicates that couples who successfully adapt to these changing roles experience improved marital satisfaction, whereas those who struggle with role adjustments often face increased marital tension (Akinwale and Omotosho, 2021; Bello and Adegoke, 2023; Okafor and Nwachukwu, 2025).

Furthermore, globalisation has influenced conflict resolution strategies and family decision-making processes among married adults. Traditionally, Nigerian families relied on extended family members, elders, and community leaders to mediate marital conflicts and maintain family harmony. However, globalisation has introduced alternative conflict resolution mechanisms such as professional counselling, online relationship resources, and self-help platforms. These modern approaches encourage individual expression, open communication, and emotional transparency, which may differ from traditional norms that emphasise endurance and collective decision-making. While some families benefit from these new approaches, others experience tension due to cultural differences between traditional and modern conflict resolution styles. Studies suggest that exposure to global media and modern ideologies has contributed to increased assertiveness and individualism among married adults, which may influence marital stability and family cohesion (Olatunji and Salami, 2022; Adeyemi and Lawal, 2024; Ibrahim and Yusuf, 2025).

Another important dimension of globalisation is its impact on family cohesion and emotional bonding. Economic globalisation has increased labour mobility, international migration, and urbanisation, resulting in physical separation among family members. Many married adults now relocate for employment opportunities, leaving spouses and children behind. While remittances and improved income may enhance family welfare, prolonged separation may reduce emotional intimacy and family unity. In addition, exposure to global lifestyles and consumer culture may create unrealistic expectations and financial pressures within families. These pressures may lead to increased stress, reduced family time, and strained relationships among married adults. Empirical studies have shown that global economic pressures and lifestyle changes significantly influence family cohesion and marital satisfaction in contemporary Nigerian society (Adebola and Ogunyemi, 2021; Ekanem and Udo, 2023; Olanrewaju and Adebisi, 2026).

Despite the growing influence of globalisation on family systems, married adults are increasingly adopting coping strategies to manage these changes. These strategies include adopting new communication techniques, renegotiating family roles, maintaining cultural traditions, and seeking support from extended family members and community networks. Some couples intentionally create time for family bonding, limit technology use, and promote shared decision-making to maintain relationship stability. Others seek professional counselling and family therapy to navigate the challenges associated with globalisation. These adaptive strategies demonstrate the resilience of Nigerian families in responding to global changes while maintaining cultural values and family cohesion (Adebayo and Ojo, 2022; Mohammed and Bello, 2024; Nwankwo and Adeyinka, 2025).

Although globalisation has received considerable scholarly attention globally, there remains limited empirical research focusing specifically on how globalisation influences family relationships and dynamics among married adults in Lagos State, Nigeria. Most existing studies have focused on general family changes, youth behaviour, migration, or gender roles without comprehensively examining communication patterns, role expectations, conflict resolution, and coping strategies within marital relationships. Furthermore, previous studies have largely relied on either quantitative or qualitative approaches, with limited integration of both methods. This creates a gap in understanding the multifaceted impact of globalisation on married adults within Nigeria’s socio-cultural context (Akinyemi and Ojo, 2022; Salawu and Ibrahim, 2023; Adegbite and Lawal, 2025).

Married adults around the world are navigating the sweeping changes brought about by globalisation, which has significantly reshaped family relationships and dynamics in contemporary societies. One major way couples are adapting is through the development of new communication strategies that accommodate changing work patterns and lifestyle demands. Global economic restructuring has increased work mobility, remote employment, and long working hours, often reducing the amount of time couples spend physically together. As a result, maintaining emotional closeness despite physical separation has become increasingly important for sustaining marital relationships. Many married adults now rely on digital communication platforms such as video calls, instant messaging, emails, and shared online calendars to maintain regular contact and coordinate family responsibilities. These technologies have made it easier for couples to stay connected across distances, strengthen emotional bonds, and manage household tasks collaboratively despite geographical separation. However, while digital communication enhances accessibility, it may also reduce face-to-face interactions, which remain essential for deeper emotional connection and relationship satisfaction (Adebayo and Oladipo, 2022; Ibrahim and Yusuf, 2023; Okeke and Nwachukwu, 2024).

Furthermore, globalisation has increased labour migration and transnational family arrangements, which have transformed how married couples maintain intimacy and emotional support. Many spouses now relocate to urban centres or foreign countries in search of better employment opportunities, creating long-distance marital relationships. In such situations, couples develop intentional communication routines such as scheduled virtual meetings, daily check-ins, and shared digital experiences to maintain emotional closeness. These adaptive communication strategies help reduce feelings of loneliness, insecurity, and emotional detachment that may arise from prolonged separation. However, studies have also indicated that long-distance marriages influenced by globalisation may experience increased trust issues, communication breakdowns, and emotional strain if communication is inconsistent or ineffective. Therefore, while technology provides opportunities for maintaining relationships, it also requires couples to develop strong communication skills, emotional intelligence, and mutual understanding to sustain marital stability (Adebola and Ojo, 2021; Bello and Akinwale, 2023; Nwankwo and Adeyinka, 2025).

In addition to technological adaptations, married partners are increasingly renegotiating role expectations within the household as globalisation continues to reshape economic and social realities. Traditionally, many societies, particularly in developing countries like Nigeria, operated under clearly defined gender roles where men were responsible for financial provision while women handled domestic responsibilities and childcare. However, globalisation has expanded access to education and employment opportunities for women, leading to increased participation of both spouses in the workforce. This shift has necessitated a more equitable distribution of household responsibilities, including childcare, financial planning, and domestic chores. Couples are increasingly adopting flexible arrangements where responsibilities are shared based on availability and competence rather than traditional gender expectations. While this adjustment may initially create tension, it often fosters mutual respect, cooperation, and stronger marital partnerships when effectively managed (Ogunleye and Ajayi, 2022; Adeyemi and Lawal, 2024; Mohammed and Bello, 2025).

Moreover, the renegotiation of family roles has also influenced decision-making processes and power dynamics within marriages. Globalisation has exposed couples to modern ideals of equality, partnership, and shared leadership within families. As a result, many married adults now engage in joint decision-making regarding finances, career choices, child-rearing, and family planning. This collaborative approach promotes transparency, trust, and emotional support within marital relationships. However, in societies where traditional values remain strong, these changes may create conflicts between modern expectations and cultural norms. Some couples experience disagreements as they adjust to shared authority and responsibility, particularly where one partner struggles to adapt to changing expectations. Despite these challenges, research indicates that couples who successfully negotiate these evolving roles tend to experience improved marital satisfaction, stronger emotional bonding, and enhanced family stability in the face of global changes (Okafor and Nwachukwu, 2023; Salami and Ibrahim, 2024; Akinyemi and Olatunji, 2026).

Therefore, this study seeks to fill this gap by investigating the impact of globalisation on family relationships and dynamics among married adults in Lagos State, Nigeria using a mixed-methods approach. The study specifically examines the positive and negative effects of globalisation on family relationships, as well as coping strategies adopted by married adults to manage these changes. By providing empirical evidence from both quantitative and qualitative perspectives, this study contributes to existing literature and offers practical implications for counselling practices, family policy development, and marital intervention programmes in Nigeria.

## Statement of the problem

Globalisation has brought rapid social, economic, and technological changes that have significantly influenced family relationships and marital dynamics across the world. In recent years, increased urbanisation, labour migration, and technological advancement have altered traditional family structures, particularly in developing countries such as Nigeria. For instance, recent reports indicate that urbanisation in Nigeria has risen from approximately 48% in 2010 to over 54% in 2023, leading to increased mobility and reduced extended family interactions that traditionally supported marital stability (World Bank, 2023). As more married adults relocate to urban centres for employment opportunities, couples increasingly experience physical separation, work-related stress, and reduced family time, all of which can influence marital relationships and family cohesion.

Furthermore, globalisation has contributed to the growing trend of dual-income households, where both spouses participate in the workforce. According to International Labour Organization (2022), female labour force participation in Sub-Saharan Africa has increased significantly, with Nigeria recording over 48% participation among married women. While this shift improves household income and promotes gender equality, it has also introduced challenges related to work-family balance, role conflict, and parenting responsibilities. Married couples now struggle to balance professional commitments with family obligations, which may lead to reduced communication, emotional strain, and marital dissatisfaction. These changes are particularly noticeable in metropolitan areas such as Lagos State, where long working hours, traffic congestion, and demanding work environments further reduce opportunities for family interaction.

In addition, technological advancements associated with globalisation have transformed communication patterns among married adults. The increasing use of smartphones, social media, and digital communication platforms has altered how couples maintain relationships. According to the National Bureau of Statistics (2024), over 60% of Nigerian adults actively use internet-enabled devices, with a significant proportion relying on digital communication for family interaction. While these technologies help couples maintain contact despite busy schedules and geographical separation, excessive reliance on digital communication may reduce face-to-face interaction and emotional intimacy. Studies have also shown that increased screen time among married couples is associated with communication breakdown, misunderstandings, and reduced relationship satisfaction in some households.

Moreover, globalisation has contributed to increased labour migration and transnational family arrangements among married adults. Reports from the International Organization for Migration (2023) indicate that Nigeria remains one of the leading countries in Africa experiencing both internal and international migration for economic opportunities. Many married adults relocate within or outside the country, leaving their spouses behind. Although migration may improve financial stability, prolonged separation may weaken emotional bonds, increase trust issues, and create parenting challenges. These realities have raised concerns about the stability of family relationships and marital satisfaction among married adults in contemporary Nigerian society.

Despite the growing influence of globalisation on family relationships, there is limited empirical research examining how these changes specifically affect married adults in Lagos State, Nigeria. Most previous studies have focused on youth behaviour, migration trends, or gender equality without providing comprehensive insights into marital relationships and family dynamics. Furthermore, many studies rely solely on quantitative or qualitative approaches, limiting a deeper understanding of the lived experiences of married adults. Therefore, there is a need for a mixed-methods study that investigates the impact of globalisation on family relationships and dynamics among married adults in Lagos State, Nigeria. This study seeks to fill this gap by providing empirical evidence that will guide counselling interventions, family policy development, and marital support programmes aimed at strengthening family relationships in the face of globalisation. Key research questions include:

1. How has Globalisation positively influenced family relationships and dynamics among married adults in Lagos State, Nigeria?
2. What are the negative effects of Globalisation on family relationships and dynamics among married adults in Lagos State?
3. What strategies have married adults in Lagos State adopted to cope with the changes brought about by Globalisation?

## Methodology

This study employed a mixed-methods research design, integrating both quantitative and qualitative approaches to provide a comprehensive understanding of the research problem. The mixed-methods approach was selected to leverage the strengths of both methodologies, allowing for numerical data analysis through surveys while also capturing in-depth insights through interviews. This approach was considered appropriate because the impact of globalisation on family relationships and dynamics involves both measurable trends and lived experiences that require deeper exploration. The study was conducted among married adults in Lagos State, Nigeria, utilising descriptive and inferential statistics for quantitative data and thematic analysis for qualitative data. The integration of both methods enhanced the robustness of findings and allowed for triangulation of results, thereby improving the credibility and validity of the study.

The quantitative phase involved a cross-sectional survey of 300 married adults in Lagos State. Participants were selected using a multi-stage sampling technique to ensure representation across different demographic groups. First, Lagos State was stratified into five key Local Government Areas (LGAs) to account for urban and semi-urban variations. Within each LGA, systematic random sampling was used to select households, and one married adult per household was invited to participate. This sampling procedure ensured that respondents were drawn from diverse socio-economic and demographic backgrounds, thereby enhancing the generalizability of the findings. The sampling procedure adopted for this study was designed to ensure adequate representation of married adults across diverse socio-economic and geographical contexts within Lagos State. Lagos State was purposively stratified into five Local Government Areas (LGAs)—Ikeja, Surulere, Alimosho, Eti-Osa, and Ikorodu—to reflect variations in urbanisation, economic activities, and population density. These LGAs were selected because they represent key socio-demographic zones within the state, including highly urbanised, semi-urban, and densely populated residential areas. This stratification enhanced the diversity of the sample and improved the generalizability of the findings.

According to available population estimates, Lagos State has a population exceeding 20 million people, with a substantial proportion comprising married adults. Given the large population size, a sample of 300 respondents was considered adequate based on standard sampling guidelines for social science research, which suggest that a sample size of 200–400 is sufficient for achieving representativeness in large populations when appropriate sampling techniques are used. The use of multi-stage sampling, including stratification and systematic random selection, further strengthened the representativeness of the sample by reducing sampling bias and ensuring proportional inclusion of participants across selected LGAs.

Following the survey, a qualitative phase was conducted to explore participants’ experiences in greater depth. Thirty participants were purposively selected from the initial survey respondents to ensure diversity in age, gender, marital duration, and socio-economic status. The selection criteria aimed to capture varied perspectives, including those in both stable and strained marriages. Semi-structured in-depth interviews were conducted either face-to-face or via video calls, depending on participant preference and availability. Each interview lasted between 45 and 60 min and was audio-recorded with participants’ consent. The qualitative phase provided deeper insights into how globalisation influences communication patterns, family roles, and coping strategies among married adults.

To enhance the robustness of findings, the quantitative and qualitative data were integrated during the interpretation phase. Survey results provided a broad overview of trends, while interview data offered deeper contextual explanations of the findings. This triangulation strengthened the validity of the study by corroborating findings across different data sources and provided a more comprehensive understanding of the impact of globalisation on family relationships and dynamics.

## Instrumentation

The researcher-designed instrument entitled Impact of Globalisation on Family Relationship and Dynamics Questionnaire (IGFRDQ) was used to collect data from the respondents. The instrument comprised five sections labelled Sections A to E. Section A collected demographic data of respondents, including age, gender, marital duration, educational level, and employment status. Sections B, C, and D each contained five items measuring different dimensions of the study, namely positive influence of globalisation, negative effects of globalisation, and coping strategies adopted by married adults. The items were generated following a thorough review of relevant literature to ensure comprehensive coverage of the constructs being measured. The questionnaire employed a four-point Likert-type scale consisting of Strongly Agree, Agree, Disagree, and Strongly Disagree, scored from 4 to 1, respectively.

To establish the validity of the instrument, the questionnaire was reviewed by five experts from the Department of Educational Guidance and Counselling, Faculty of Education, University of Ilorin. Their feedback was used to refine and improve the clarity, relevance, and appropriateness of the items, thereby ensuring face and content validity. In addition, construct validity was strengthened by ensuring that items aligned with the research objectives and conceptual framework of the study.

The reliability of the instrument was established using a test–retest reliability procedure. The questionnaire was administered to a pilot group of respondents outside the study area on two occasions separated by a two-week interval. The scores obtained were correlated using Pearson’s correlation coefficient, yielding a reliability coefficient of 0.89, which indicates a high level of stability over time. In addition, internal consistency of the instrument was assessed using Cronbach’s alpha coefficient. The overall Cronbach’s alpha value of 0.87 confirmed that the instrument items were consistently measuring the constructs of interest.

Section E of the instrument included open-ended questions aimed at providing deeper qualitative insights into respondents’ experiences. Sample questions included: “Can you describe a way globalisation has helped improve your relationship with your spouse or children?”; “What challenges has globalisation introduced in your family life?”; and “What coping mechanisms have you and your spouse adopted to manage these changes?” These open-ended responses complemented the quantitative data and enriched the overall findings of the study.

Quantitative data collected from Sections A to D were analysed using descriptive and inferential statistics. Descriptive statistics such as frequency counts, percentages, mean scores, and standard deviation were used to summarise demographic information and responses. In addition, inferential statistics such as correlation analysis and regression analysis were conducted to examine relationships between globalisation and family relationship dynamics among married adults. These analyses strengthened the robustness of the findings and addressed previous methodological limitations identified in the review.

Qualitative responses from Section E and interview data were subjected to thematic analysis. The researcher carefully reviewed responses, identified recurring patterns, and categorised them into themes relevant to the research objectives. The themes were then interpreted and integrated with the quantitative findings to provide a comprehensive understanding of the impact of globalisation on family relationships and dynamics among married adults in Lagos State, Nigeria.

## Results

### Participants

The demographic distribution of the 300 married adults in Lagos State reveals key insights into the sample. A majority of respondents are middle-aged, with 30% between 30 and 39 years and 33.3% between 40 and 49 years, reflecting individuals in their prime family-raising years. The gender distribution is nearly balanced, with 50% male and 48.3% female, alongside 1.7% identifying as “Other,” providing a diverse range of perspectives. Most respondents have been married for 5–14 years, with 26.7% married for 10–14 years, indicating substantial marital experience. Educationally, the sample is well-educated, with 30% holding a HND or bachelor’s degree and 16.7% having a master’s degree, suggesting exposure to global influences. Employment status also indicates economic activity, with 40% employed full-time and 26.7% self-employed, suggesting a strong connection to global economic trends. Only 10% reported being unemployed, with smaller proportions being retired (6.7%) or students (3.3%). Overall, this sample reflects a group of middle-aged, educated, and economically active individuals with established marriages, making them well-suited to provide insights into the impact of Globalisation on family dynamics.

Research question one: How has Globalisation positively influenced family relationships and dynamics among married adults in Lagos State, Nigeria?

Table 1 reveals the grand mean score of 3.54 which is greater than the mean cut-off point of 2.50. Specifically, the highest mean scores (3.60) were recorded on three items: enhanced communication via modern technology, positive influence of global media in managing family challenges, and strengthened family bonds through access to global parenting resources. This implies that access to information, improved communication technologies, and exposure to diverse family and parenting practices across cultures have significantly contributed to family development. On the same note, the relatively high mean scores on items relating to cultural appreciation (3.40) and equality in decision-making (3.50) suggest that Globalisation fosters more balanced relationships and mutual respect within households. This indicates that the respondents agreed that Globalisation has had a positive influence on family relationships and dynamics. The positive impacts include increased access to information, improved communication, and exposure to diverse cultural values.

**Table 1:** Mean and standard deviation showing respondents’ expression on the positive influence of globalisation.

S/N	Statement	Mean	SD (SD)
1	Globalisation has enhanced communication between me and my spouse through modern technology.	3.6	0.52
2	Exposure to global cultures has improved our understanding and appreciation for each other.	3.4	0.41
3	Global media has positively influenced how we manage family challenges.	3.6	0.50
4	Access to global parenting resources has strengthened our family bond.	3.6	0.43
5	Globalisation has contributed to more equality in decision-making between my spouse and me.	3.5	0.53
	Grand Mean	3.54	

In order to get deeper qualitative insights into the experiences of the married adults in Lagos State on the positive influence of globalisation, 30 married adults were interviewed. The question and responses were as follows:

Question “Can you describe a way Globalisation has helped improve your relationship with your spouse or children?”

The following are the themes and the response to the question:

1 Enhanced Communication and Connectivity
o Respondent 1: “Globalisation has made it easier for us to stay in touch with family overseas, so we can communicate more frequently and share our lives with relatives, which strengthens our bond.”
o Respondent 4: “The ability to video call family members across the globe has helped my children feel more connected to their extended family, even though we live far apart.”
o Respondent 6: “Thanks to Globalisation, my spouse and I can easily access online therapy sessions together, which has improved our communication and understanding.”
o Respondent 8: “I can work remotely now, which has allowed me to spend more time with my spouse and children, strengthening our bond through shared experiences.”
o Respondent 22: “My spouse and I can now connect online for date nights or casual chats even when we are apart for work, making our long-distance relationship feel much more intimate.”
o Respondent 28: “The ease of online communication has allowed my spouse and I to stay emotionally connected during times when work or travel separates us, ensuring that we maintain our closeness.”
2 Cultural Exposure and Understanding
o Respondent 3: “I think the exposure to different cultures through media has helped me and my spouse appreciate our differences and become more tolerant of each other’s perspectives.”
o Respondent 7: “My children have online friends from different countries, and it has helped them become more open-minded and accepting of diverse cultures, which enriches our family dynamic.”
o Respondent 11: “Globalisation has exposed us to a wide range of cultural traditions and ideas, which has made us more empathetic and understanding as a family, especially when it comes to different cultures and backgrounds.”
o Respondent 21: “We enjoy learning about global celebrations, and celebrating these diverse traditions has brought our family closer together.”
o Respondent 24: “Global exposure to different parenting styles has helped me adjust my approach to parenting, making it more balanced and effective, which has improved our family dynamics.”
3 Learning and Educational Opportunities
o Respondent 2: “Through global online learning resources, my children have access to educational content from around the world, which has sparked interesting conversations between us.”
o Respondent 7: “My children are exposed to a variety of global learning platforms, and we now have more enriching educational discussions as a family.”
o Respondent 19: “The exposure to global ideas on work-life balance has helped me and my spouse create a more harmonious home life, where we prioritise family time.”
o Respondent 20: “Having access to global educational tools has allowed me to help my children with their studies, and we now spend more quality time together learning.”
o Respondent 23: “My children are learning from international perspectives, which has helped them engage in thoughtful discussions about the world, making family conversations more interesting.”
o Respondent 27: “The variety of foods and recipes available through global platforms has allowed my family to explore new meals, creating fun family activities around cooking together.”
4 Shared Experiences and Family Bonding
o Respondent 5: “Globalisation has introduced us to different cuisines and traditions, which we enjoy exploring together as a family, making our meals more meaningful and fun.”
o Respondent 12: “Through global sports events, my children and I bond by watching games from different countries, and it has become a fun family tradition.”
o Respondent 17: “Through international collaboration opportunities, my spouse and I both grew professionally and personally, which has made our relationship stronger and more fulfilling.”
o Respondent 25: “We enjoy watching international travel vlogs together, which has sparked discussions about potential family trips and strengthened our bond as we dream about future adventures.”
o Respondent 29: “We’ve become more involved in global causes, and doing charity work together has brought our family closer, giving us a shared purpose.”
5 Improved Parenting and Family Dynamics
o Respondent 6: “Thanks to Globalisation, my spouse and I can easily access online therapy sessions together, which has improved our communication and understanding.”
o Respondent 9: “Globalisation has introduced us to a wide variety of parenting techniques from around the world, and we have been able to use some of them to improve how we communicate with our children.”
o Respondent 24: “Globalisation has exposed us to a wide variety of parenting techniques, and I’ve been able to use some of them to improve how I relate to my children.”
o Respondent 26: “Thanks to Globalisation, I can join online support groups for parenting, which has provided valuable advice and support that has helped me be a better parent.”
6 Work-Life Balance and Family Time
o Respondent 8: “I can work remotely now, which has allowed me to spend more time with my spouse and children, strengthening our bond through shared experiences.”
o Respondent 18: “The exposure to global ideas on work-life balance has helped me and my spouse create a more harmonious home life, where we prioritise family time.”
o Respondent 19: “The exposure to global ideas on work-life balance has helped me and my spouse create a more harmonious home life, where we prioritise family time.”
7 Travel and Adventure
o Respondent 14: “Globalisation has allowed me and my spouse to connect more deeply by making travel more affordable and accessible, allowing us to explore new places together.”
o Respondent 21: “We enjoy learning about global celebrations, and celebrating these diverse traditions has brought our family closer together.”
o Respondent 25: “We enjoy watching international travel vlogs together, which has sparked discussions about potential family trips and strengthened our bond as we dream about future adventures.”
8 Emotional Support and Relationship Strengthening
o Respondent 6: “Thanks to Globalisation, my spouse and I can easily access online therapy sessions together, which has improved our communication and understanding.”
o Respondent 22: “My spouse and I can now connect online for date nights or casual chats even when we are apart for work, making our long-distance relationship feel much more intimate.”
o Respondent 28: “The ease of online communication has allowed my spouse and I to stay emotionally connected during times when work or travel separates us, ensuring that we maintain our closeness.”
9 Exposure to Global Entertainment
o Respondent 12: “Through global sports events, my children and I bond by watching games from different countries, and it has become a fun family tradition.”
o Respondent 18: “Globalisation has brought new forms of entertainment, such as international films and music, that we enjoy as a family and help us connect with one another.”
o Respondent 25: “We enjoy watching international travel vlogs together, which has sparked discussions about potential family trips and strengthened our bond as we dream about future adventures.”

In summary, this result shows that Globalisation has a positive influence on family relationships and dynamics. The responses illustrate various ways Globalisation has impacted relationships, with a common theme of enhanced communication, cultural exchange, and shared experiences.

Research question two: What are the negative effects of Globalisation on family relationships and dynamics among married adults in Lagos State?

Table 2 shows that Globalisation has led to less face-to-face time with my spouse due to busy schedules (Mean = 3.40, SD = 0.72), “Exposure to foreign cultures has sometimes caused conflicts in our values as a couple” (Mean = 3.15, SD = 0.85), “Social media has negatively impacted trust in my marriage” (Mean = 2.95, SD = 0.91), “The pursuit of global standards of living has increased financial pressure on my family” (Mean = 3.35, SD = 0.78), and “Global work demands have made it difficult to maintain close family relationships” (Mean = 3.25, SD = 0.83).

**Table 2:** Mean and standard deviation showing respondents’ expression on the negative effects of globalisation on family relationships and dynamics.

S/N	Statement	Mean	SD
6	Globalisation has led to less face-to-face time with my spouse due to busy schedules.	3.40	0.72
7	Exposure to foreign cultures has sometimes caused conflicts in our values as a couple.	3.15	0.85
8	Social media has negatively impacted trust in my marriage.	2.95	0.91
9	The pursuit of global standards of living has increased financial pressure on my family.	3.35	0.78
10	Global work demands have made it difficult to maintain close family relationships.	3.25	0.83
	Grand Mean	3.22	

The grand mean score of 3.22 indicates a general agreement among respondents that Globalisation has had adverse effects on their family life. Specifically, participants highlighted increased conflicts, especially in value systems due to cultural exposure, changing role expectations resulting from global work demands and lifestyle pressures, and a noticeable decrease in family cohesion as a result of less shared time and digital distractions. These findings suggest that while Globalisation brings opportunities, it also introduces considerable strain on marital relationships and family dynamics in the context of Lagos State.

In order to get deeper understanding on the negative effect of globalisation among married adults in Lagos State, 30 married adults were interviewed. The question and responses were as follows:

Question: “What challenges has Globalisation introduced in your family life?

Themes and responses:

1 Time and Physical Distance
o Respondents 9: “We face time zone issues with family members living abroad, making it hard to maintain close relationships.”
o Respondents 25: “Global travel has made us physically distant, and it’s difficult to keep that emotional closeness.”
o “Family gatherings are more difficult because people are often travelling for work or studying abroad.”
o Respondents 10: “My spouse’s job requires frequent international travel, which strains our relationship and family life.”
o Respondents 28: “Working long hours to compete globally has left us with little time for each other.”
o Respondents 26: “My children have become more interested in foreign cultures and languages, but sometimes it feels like they are drifting away from our own heritage.”
o Respondents 29: “We have relatives in multiple countries, and it’s challenging to stay connected due to differing lifestyles and time differences.”
2 Cultural Differences and Clashes
o Respondents 11: “The pressure to adapt to different cultures has caused some tension in the family, especially with younger members who embrace global trends.”
o Respondents 7: “The cultural differences that come with interacting with people from different countries have led to misunderstandings in our family.”
o Respondents 27: “My children are more exposed to western values, which sometimes clash with our traditional family beliefs.”
o Respondents 30: “Globalisation has introduced different expectations and pressures, especially concerning how we raise our children.”
o Respondents 8: “There’s a growing gap between family members, especially older generations who find it harder to adjust to globalised practices.”
o Respondents 24: “We’ve seen a rise in individualism within the family, as everyone becomes more focused on their global identity than on the family unit.”
3 Technology and Media Influence
o Respondents 12: “We’ve had to adjust to new technologies and social media, which has led to more distractions and less quality family time.”
o Respondents 5: “The constant flow of information from around the world can overwhelm us, affecting our mental health as a family.”
o Respondents 23: “Globalisation has made it easier to access information, but it has also increased family disagreements over what’s right and wrong.”
o Respondents 6: “Globalisation has brought a lot of new food, clothing, and technology into our lives, but it has also created tension over what is considered ‘normal’ in our family.”
4 Work and Career Pressures
o Respondents 22: “The pressure to keep up with global standards in terms of education and career success has affected the way we relate to one another.”
o Respondents 13: “The desire for a more global career means that family members are more likely to move abroad, and it disrupts family stability.”
o Respondents 4: “My spouse’s job requires frequent international travel, which strains our relationship and family life.”
5 Changing Family Dynamics and Values
o Respondents 18: “It’s harder to maintain family traditions when everyone is influenced by global trends and lifestyles.”
o Respondents 3: “Family members have different work schedules due to Globalisation, making it difficult to find time for everyone to be together.”
o Respondents 20: “The rapid pace of life due to Globalisation leaves less time for meaningful conversations at home.”
o Respondents 14: “We’ve seen a rise in individualism within the family, as everyone becomes more focused on their global identity than on the family unit.”
o Respondents 21: “Globalisation has made family relationships less cohesive as members prioritise their global identities over family connections.”
6 Generational Differences
o Respondents 2: “There’s a growing gap between family members, especially older generations who find it harder to adjust to globalised practices.”
o Respondents 15: “The desire to keep up with global trends has caused friction between younger and older generations in our family.”
7 Materialism and Consumerism
o Respondents 17: “The availability of international products has led to family members becoming more materialistic and demanding.”
o Respondents 19: “Globalisation has introduced new consumer behaviours that sometimes conflict with our family’s values.”
8 Social and Psychological Effects
o Respondents 1: “The pressure to conform to global beauty standards has affected our self-esteem, especially for younger family members.”
o Respondents 16: “It’s tough to maintain a work-life balance because the global economy demands more time and energy from each of us.”

In summary, the respondents also attested that globalisation has negative impact on family relationships and dynamics. These themes capture the various dimensions of Globalisation’s impact on family life, from emotional and social challenges to practical adjustments related to time, work, and values.

Research question three: What strategies have married adults in Lagos State adopted to cope with the changes brought about by Globalisation?

As shown in Table 3, the item with the highest mean score (*M* = 3.52, SD = 0.65) indicates that many respondents strongly agreed or agreed that they had adopted new communication strategies to stay connected with their spouses. Additionally, respondents reported frequently re-negotiating marital roles in response to global influences (*M* = 3.34, SD = 0.72), and relying on extended family and community networks during times of stress (*M* = 3.41, SD = 0.69). The item on incorporating cultural traditions also received a relatively high mean score (*M* = 3.47, SD = 0.63), suggesting that while adapting to Globalisation, families still valued and maintained cultural practices as a source of stability. The use of family meetings and open discussions to address change was also evident (*M* = 3.28, SD = 0.75).

**Table 3:** Mean and standard deviation showing coping strategies adopted by married adults in Lagos State.

S/N	Statement	Mean	S.D
11	My spouse and I have adopted new communication strategies to stay connected.	3.52	0.65
12	We have re-negotiated our marital roles to adapt to modern global influences.	3.34	0.72
13	We rely on support from our extended family and community during stressful times.	3.41	0.69
14	We use family meetings or open discussions to address changes brought by Globalisation.	3.28	0.75
15	We intentionally incorporate cultural traditions to maintain stability within the family.	3.47	0.63

Overall, these findings suggest that married adults in Lagos State are adapting to Globalisation through a combination of modern and traditional strategies, including the use of improved communication, flexibility in role expectations, community support, and cultural preservation.

In order to get deeper understanding on coping mechanism of married adults in Lagos State on the negative impact of globalisation on family relationships and dynamic, 30 married adults were also interviewed. The question and responses were as follows:

Question: What coping mechanisms have you and your spouse adopted to manage these changes?

1 Communication
o Respondent 2: “Communication has been key, so we make sure to talk openly about how we are feeling.”
o Respondent 9: “We talk to each other more frequently about our emotions and have become more supportive.”
o Respondent 13: “We have regular check-ins where we talk about what’s working and what’s not in our relationship.”
o Respondent 26: “We talk through difficult decisions together, and we avoid making major choices without each other’s input.”
2 Shared Activities/Hobbies
o Respondent 1: “We’ve started scheduling regular date nights to reconnect and discuss our feelings.”
o Respondent 10: “We adopted a shared hobby, like gardening, to bond and take our minds off stress.”
o Respondent 17: “We make time for regular walks or hikes together to clear our minds.”
o Respondent 12: “We started cooking meals together, which has brought us closer.”
3 Mindfulness and Stress Relief
o Respondent 5: “My spouse and I practice mindfulness to help stay present and calm in the moment.”
o Respondent 4: “We’ve started exercising together, whether it’s walking or going to the gym, to relieve stress.”
o Respondent 23: “We take breaks from social media to focus on each other without distractions.”
4 Emotional and Psychological Support
o Respondent 16: “We rely on our extended family for support when things get tough.”
o Respondent 18: “We both journal about our thoughts and share our entries with each other to foster empathy.”
o Respondent 11: “Therapy has been a big help for both of us to work through our individual and collective challenges.”
o Respondent 27: “We’ve built a strong support network with close friends to lean on when needed.”
5 Maintaining Intimacy
o Respondent 20: “We’ve prioritised our intimacy, making sure we spend quality time alone together.”
o Respondent 21: “We’ve become more patient and understanding, especially when the other person is stressed.”
6 Positive Reinforcement and Patience
o Respondent 14: “We use positive affirmations with each other to stay motivated and optimistic.”
o Respondent 19: “We’ve been making an effort to surprise each other with small acts of kindness.”
o Respondent 15: “Our coping mechanism has been dividing tasks more equally to reduce overwhelm.”
o Respondent 22: “My spouse and I have started a gratitude practice where we reflect on the good things in our life.”
o Respondent 25: “We’ve learned to ask for help from each other more frequently and to accept it.”
7 Individual Space and Balance
o Respondent 8: “We take time apart occasionally to recharge individually, which helps us be better together.”
o Respondent 24: “We’ve worked on fostering a sense of humour to navigate through difficult situations.”
o Respondent 28: “We try to maintain a routine, as it helps reduce anxiety and creates stability.”
8 Routine and Structure
o Respondent 28: “We try to maintain a routine, as it helps reduce anxiety and creates stability.”
o Respondent 7: “We try to maintain a healthy work-life balance by setting boundaries with work-related tasks.”
9 Humour
o Respondent 6: “We’ve leaned on humour to help us cope with tough situations.”
o Respondent 24: “We’ve worked on fostering a sense of humour to navigate through difficult situations.”
10 Self-Care
o Respondent 29: “We’ve been focusing on our spirituality together, which has helped us stay grounded.”
o Respondent 30: “We’ve learned to give each other space when we need it and return to communication when ready.”

In summary, these themes capture the various ways couples adapt to changes and cope together, highlighting the importance of communication, shared activities, emotional support, intimacy, and personal wellbeing.

Table 4 shows the Pearson correlation analysis between globalisation and family relationship dynamics. The result revealed a significant positive relationship between globalisation and family relationship dynamics (*r* = 0.62, *p* < 0.05). This indicates that changes associated with globalisation are strongly related to variations in family interactions among married adults in Lagos State.

**Table 4:** Pearson correlation showing relationship between globalisation and family relationship dynamics.

Variables	Globalisation	Family relationship dynamics
Globalisation	1.00	
Family relationship dynamics	0.62^*^	1.00

* *p* < 0.05.

Table 5 presents the regression analysis examining the predictive influence of globalisation on family relationship dynamics. The results indicate that globalisation significantly predicts family relationship outcomes (*β* = 0.58, *t* = 9.21, *p* < 0.05). The model explains approximately 34% of the variance in family relationship dynamics (*R*^2^ = 0.34), suggesting that globalisation is a significant determinant of changes in family interactions among married adults in Lagos State.

**Table 5:** Regression analysis showing the predictive influence of globalisation on family relationship dynamics.

Model variables	*B*	Std. Error	Beta (*β*)	*t*-value	Sig. (*p*)
Constant	1.12	0.21	—	5.33	0.000
Globalisation	0.58	0.06	0.58	9.21	0.000^*^

*R* = 0.62, *R*^2^ = 0.34, Adj. *R*^2^ = 0.33, ^*^p < 0.05.

## Discussion

The finding of the study revealed that globalisation has positive impact on the family relationships and dynamics. It positive impacts include increased access to information, improved communication, and exposure to diverse cultural values. This implies that Globalisation, through technological advancements and cultural exchange, enables families to stay connected, share knowledge, and adopt progressive values that strengthen familial bonds. This could be that digital communication tools such as social media, video calls, and messaging apps have revolutionised long-distance family interactions, making it easier for members to stay connected and that exposure to global cultures promotes tolerance and flexibility within families, as they integrate beneficial aspects of different traditions while maintaining core values. The finding supports the study of Schweitzer et al. (2019) who found that migrant families maintain strong emotional bonds through digital communication, mitigating the negative effects of physical separation. Similarly, Madianou and Miller (2020) found how transnational families use technology to sustain intimacy and shared decision-making. Arnett (2018) observed that exposure to global cultures enhances familial adaptability, particularly among younger generations who blend traditional and modern values.

The study’s finding also revealed that globalisation has negative impacts on family relationships and dynamics which include increased conflict, changing role expectations, and decreased family cohesion. This suggests that the interconnectedness of the world has created new stressors that families may struggle to manage. The reasons behind these negative impacts stem from the broader societal changes brought about by Globalisation. For instance, greater mobility, access to digital technologies, and the influence of global culture can create competing demands on family members’ time and priorities. These external pressures may lead to tensions within families as they navigate changing expectations of gender roles, responsibilities, and work-life balance. Research by Leung et al. (2022) aligns with this finding, as their study reveals that Globalisation leads to families experiencing more conflict due to mismatched expectations and the challenges of balancing modern, individualistic goals with traditional family values. Furthermore, a study by Ibarra et al. (2018) indicates that while Globalisation can provide economic opportunities, it can also undermine family cohesion as members prioritise work and personal goals over family togetherness.

The findings also revealed that married adults in Lagos State are adapting to these changes by adopting new communication strategies, re-negotiating role expectations, and seeking support from extended family and community networks. The finding suggests that married adults are employing adaptive strategies to mitigate the negative effects of Globalisation on family relationships and dynamics. This could be that Globalisation, characterised by increased mobility, technological advancements, and economic pressures, has disrupted traditional family structures, leading to challenges such as emotional distance, role conflicts, and work-family imbalances and these made married adopt to adopt various coping mechanism. The finding relates to the study of Hochschild and Machung (2012) who asserted in order to adopt to new technological era, married partners are increasingly re-negotiating role expectations within the household. Beck-Gernsheim (2002) reported that the strain introduced by Globalisation has prompted many families to seek emotional and practical support from extended family members and community networks.

Beyond empirical alignment, the findings of this study extend existing knowledge by demonstrating that globalisation does not merely influence family structures but actively reshapes relational processes within marriages. Specifically, the study highlights the coexistence of collectivist and individualistic orientations within Nigerian families, suggesting a hybridization of family systems rather than a complete erosion of traditional values. This contributes to sociological discourse by showing that globalisation produces adaptive, rather than purely disruptive, transformations in family life. Thus, the findings refine existing perspectives by emphasising the agency of families in negotiating global pressures while maintaining cultural continuity.

## Conclusion

It was concluded that globalisation has significantly impacted family relationships and dynamics among married adults in Lagos State, Nigeria. While there are benefits to globalisation, such as increased access to information and improved communication, there are also challenges, including increased conflict and changing role expectations. Despite these challenges, married adults in Lagos State are adapting by embracing new communication methods, redefining traditional roles, and drawing support from extended family and community networks.

### Sociological and practical implications

The findings of this study have important sociological implications for understanding family transformation in a globalised context. The results indicate a shift from traditional collectivist family structures towards more flexible and hybrid systems that integrate both local and global influences. This suggests that family institutions in Nigeria are evolving in response to economic, technological, and cultural changes associated with globalisation. At a societal level, the increasing adoption of dual-income structures, changing gender roles, and digital communication patterns reflects broader structural transformations in social organisation. These changes call for policies that support work-life balance, family cohesion, and social support systems. In practical terms, while counselling remains relevant, there is also a need for community-based and policy-driven interventions that address the broader social pressures affecting families. Strengthening social institutions, promoting cultural continuity, and supporting family-friendly work environments are critical for sustaining family stability in a globalised society.

### Recommendations

Based on the findings of the study, it was recommended that:

1. Married adults, policymakers, and community leaders should work together to promote healthy family relationships and dynamics in the face of globalisation. This can be achieved by providing education and training on effective communication, conflict resolution, and role negotiation, as well as promoting community-based initiatives that support family cohesion and resilience.
2. Marriage counsellors should provide family counselling and mediation services that help families manage and resolve conflicts stemming from differing worldviews or cultural expectations.

## Data Availability

The original contributions presented in the study are included in the article/supplementary material, further inquiries can be directed to the corresponding author.
